# Bioavailability and Bioactivity of Encapsulated Phenolics and Carotenoids Isolated from Red Pepper Waste

**DOI:** 10.3390/molecules24152837

**Published:** 2019-08-05

**Authors:** Jelena Vulić, Vanja Šeregelj, Ana Kalušević, Steva Lević, Viktor Nedović, Vesna Tumbas Šaponjac, Jasna Čanadanović-Brunet, Gordana Ćetković

**Affiliations:** 1 Faculty of Technology, University of Novi Sad, Bulevar cara Lazara 1, 21 000 Novi Sad, Serbia; 2 Faculty of Agriculture, University of Belgrade, Nemanjina 6, 11 080 Zemun, Serbia; 3 Institute of Meat Hygiene and Technology, Kaćanskog 13, 11 040 Belgrade, Serbia

**Keywords:** red pepper waste, encapsulation, bioavailability, digestion

## Abstract

In order to deactivate the health properties of bioactive compounds, they need to withstand the effects of food processing, their potential release from the food matrix, and remain bio-accessible in the gastrointestinal tract. Bio-actives from different plants are prone to oxidative degradation, and encapsulation is an effective method in improving their stability. In the present study, red pepper waste (RPW), a by-product of vegetable processing industry, was encapsulated in whey protein using spray and freeze-drying techniques. The aim was to evaluate the effects of in vitro gastrointestinal digestion on the release and bioactivity of encapsulated bio-actives, after each digestion step. The results showed that the release of phenolics and carotenoids, as well as antioxidants, anti-hyperglycemic, and anti-inflammatory activities are influenced by pH and intestinal fluid, with pH 7.5 exhibited at higher levels. There was a rapid initial release of carotenoids from whey protein matrices, while a more gradual increase of phenolics was observed, reaching around 50% for both encapsulates first at 6 h and 37 °C, and small intestine conditions. The encapsulation of RPW demonstrated a protective effect against pH changes and enzymatic activities along digestion, and contributed to the increase in bio-accessibility in the gut. Also, the results suggest that encapsulation is an efficient method for valorization of bio-actives from RPW, with improvements in nutrition, color, and bioactive properties.

## 1. Introduction

Recent research efforts have focused on exploring natural bioactive compounds and their capacity, to prevent and inhibit multifaceted health disorders arising from the overproduction of free-radical species [1]. The excessive generation of these reactive species leads to different pathophysiological conditions by causing significant damage to proteins, lipids, nucleic acids, and significantly contributing to cardiovascular diseases, cancers, neurodegenerative disorders, and other diseases related to “oxidative stress”. Additionally, a direct correlation between oxidative stress and inflammation and insulin resistance (a key factor for type-II diabetes mellitus) were emphasized by Hussain et al. [2] and Hurlle et al. [3].

Phenolics and carotenoids represent widely distributed naturally-occurring antioxidants in plants, that exhibit many health benefits when consumed at an appropriate level. Peppers (*Capsicum annuum* L.) are native to Central and South America, and belong to the Solanaceae family [4,5]. Nutritional, health-promoting, and sensory attributes make pepper an important industrial crop with high potential for economic value, due to their versatility consumption as fresh vegetables in salads, cooked meals, and when dehydrated to make spices. During the processing of raw plant materials, large amounts of wastes are generated, and are commonly used as animal feed or deposed to landfills. However, human opinions have changed around the disposal of raw plant materials for several reasons, including the growing environmental concerns and demand for greater controls to minimize the impact of waste on human health [5]. Different studies reported that peels and seeds contain a significantly higher phenolic content than edible portions [6,7]. Also, the pericarp of the pepper fruit accumulates considerable amounts of numerous carotenoid pigments, giving the ripe fruit intensive colors, which contribute to a growing awareness of the valorization of numerous valuable compounds present in pepper wastes [8].

The bioavailability and bioactivity of present bioactive compounds are limited by their stability against oxidative degradation during storage and bio-accessibility from the food matrix during gastrointestinal digestion [9]. Encapsulation technology is an effective method aimed at improving their stability by entrapping core materials with the coating agent. The selection of the encapsulation method plays an important role in the encapsulate properties that would influence the retention of bioactive compounds before consumption and their bio-accessibility [10]. Freeze-drying is an important technique for the protection of the sensitive compounds, biological activities, color, aroma, texture, and the nutritional values of food, which compensate for its high operating costs. On the other side, spray drying is a widely used and relatively low-cost technique because of the very short time being in contact with the drying medium. Although, the high operation temperature may lead to some quality losses [11,12]. 

Among a wide range of carrier materials, whey protein isolate has been applied in this study for its significant commercial potential as a by-product of cheese production, with superior gelling and emulsification properties, rich in β-lactoglobulin and α-lactalbumin. Furthermore, it is a suitable vehicle for different bioactive compounds, based on sustained antioxidative activity throughout simulated digestion models [13,14]. Additionally, the use of this material may represent further improvement in the nutritional value of the final product. Thus, the encapsulated extract of pepper waste could be used in the food industry as a colorant in the production of dairy and meat products, potato chips, popcorn, salads, mayonnaise, soups, sauces, jams, beverages, and bakery additives [15]. In vitro simulated digestion studies have become increasingly popular for predicting the bio-accessibility of bioactive compounds in the human gastrointestinal tract. For this purpose, it is necessary to demonstrate that biological molecules are released from encapsulates during the digestion process, with considerable activity to ensure its benefits in the organism.

Therefore, this study was designed to investigate the bioavailability and bioactivity of encapsulated phenolics and carotenoids, isolated from red pepper waste, during in vitro simulated gastrointestinal digestion. The in vitro digestion of freeze-dried encapsulates (FDE) and spray-dried encapsulates (SDE) was determined by simulation of digestion in gastric fluid (SGF) and intestinal fluid (SIF). The phenolic and carotenoid compounds, present in red pepper waste encapsulates, were identified and quantified to gain an insight into the compounds responsible for its health benefits. Several antioxidant assays, free radical scavenging activity on DPPH radicals (SA), reducing power (RP), β-carotene bleaching assay (BCB), as well as pharmacological activities, anti-hyperglycemic (AHgA) and anti-inflammatory activities (AIA), were estimated. Also, the experiments of bioactive compounds release from whey protein matrices over time were conducted at 37 °C in small intestine-simulated conditions. 

## 2. Results and Discussion

### 2.1. Bioavailability of Encapsulated Bioactive Compounds during in Vitro Simulated Gastrointestinal Digestion

The release of bioactive compounds was significantly affected by pH along the digestion process. These results are in accordance with the literature, which also found differences in the release of bio-actives from encapsulates exposed to simulated gastrointestinal fluids [10,14]. Since these encapsulates are designed for possible incorporation into food products, it is essential that release be controlled by the applied wall material during gastrointestinal digestion, as they preserve their biological activities and protecting them from degradation. Results of spectrophotometric analysis of total phenolics and carotenoids, as well as individual phenolics determined by HPLC analysis, before, and after, simulated in vitro gastrointestinal digestion are presented in Table 1. For all investigated compounds, the SIF with pH 7.5 exhibited higher levels (*p* < 0.05). 

Spectrophotometrical analysis revealed that after intestinal digestion of FDE and SDE, TPh values were 62.12%, and 56.88% of the initial phenolic contents, respectively, while TCar decreased to 20.4%, and 15.05% of the initial carotenoid content, respectively. The similar release profiles were obtained by Yi et al. [16] and confirmed that whey protein isolate nanoparticles are good carriers for delivering bioactive compounds, which exhibit low release profiles in gastric fluids and high release profile in intestinal fluids, although they prepared whey protein nanoparticles by a different method. Additionally, whey protein is poorly digestible by pepsin, but can be hydrolyzed rapidly by different enzymes in intestinal juice, making whey protein ideally suitable for the controlled release of bioactive compounds [17]. 

Data from HPLC analysis revealed the presence of three hydroxybenzoic acids (gallic, vanillic, and protocatehuic acid), two hydroxycinnamic acids (caffeic and chlorogenic acid), one flavan-3-ols (epicatechin), and three flavonols (rutin, quercetin, and myricetin). Similar phenolic and flavonoid profiles have also been reported in scalded Jalapeno pepper industrial by-product and some Egyptian sweet peppers [18,19]. Generally, HPLC results are in accordance with spectrophotometrical, where phenolic compounds in FDE were higher than those in SDE. During the gastric digestion 93.73% of phenolic compounds was released from FDE and 43.83% from SDE. The similar trend was observed after intestinal digestion. Hence, the release of 94.28% from FDE and 81.58% from SDE. Presented results demonstrated that phenolic compounds in FDE are highly bio-accessible in both gastrointestinal fluids. 

### 2.2. Bioactivity of Encapsulated Bioactive Compounds during in Vitro Simulated Gastrointestinal Digestion

Natural bioactive compounds, widely distributed in fruits and vegetables, are considered to exert health beneficial properties, mainly through their antioxidant activities. There is no single chemical assay that can accurately evaluate the contribution of the hydrophilic and lipophilic compounds to the total antioxidant activity of the plants [20]. Also, the antioxidant activity of these compounds might be affected by the chemical transformations resulting during gastrointestinal digestion. Therefore, several assays were performed, i.e., SA and RP with hydrophilic fractions, and BCB with lipophilic fractions. The antioxidant activity results of FDE and SDE before and after in vitro simulated gastrointestinal digestion are presented in Table 2.

The DPPH radical scavenging assay is widely used to determine the antioxidant activity of phenolic compounds, based on the capacity of stable DPPH free radicals to react with hydrogen donors. Enzymatic reactions and different pH conditions, during simulated digestion, lead to decreasing molecule size, and it was found that small molecules can better access the radical site of DPPH [21]. These could explain the increase in SA after intestinal digestion, as well as the higher phenolics amounts released in this medium than in SGF. FDE exhibited the significantly higher SA (*p* ˂ 0.05) than SDE, which is correlated with released phenolics content. Moran et al. [22] reported the effect of pH could be different for various phenolics. Phenolics represent a large group with different classes of bioactive compounds, so the differences in SA, in SGF and SIF, may be explained by the diversity of the present/released phenolics. Furthermore, the chemical structure of phenolics, i.e., number and position of hydrogen-donating hydroxyl groups on the aromatic rings, also possess a great impact on the free radical scavenging activity [23]. Ydjedd et al. [24] followed the in vitro gastrointestinal digestion of encapsulated and non-encapsulated phenolic compounds of Carob pulp extracts, and their antioxidant capacity. The most important phenolics and flavonoids, as well as antioxidant activities for encapsulated extracts, were observed in the SIF, while for non-encapsulated extracts, these properties were significantly higher in the gastric step. On the other side, Norkaew et al. [25] were studying the effect of wall materials on the release characteristics of encapsulated black rice extract. During in vitro intestinal digestion, the black rice microcapsules obtained by spray-drying with pure whey protein released high amounts of anthocyanins and total phenolic compounds, and showed a remarkable increase in the antioxidant activity.

The reducing power assay may serve significant reflection of antioxidant activity, which is based on the principle that substances, which have reduction potential, react with potassium ferricyanide (Fe^3+^) to form potassium ferrocyanide (Fe^2+^), which then reacts with ferric chloride to form ferric–ferrous complex with an absorption maximum at 700 nm. Regarding the RP of encapsulated RPW extract, both FDE and SDE showed significant differences (*p* ˂ 0.05) during the gastrointestinal digestion. The RP increased during the digestion process where the higher activity was recorded for the FDE in the SIF. However, these values are lower than the initial non-digested samples. The similar trend was observed by Stanisavljevic et al. [26] who investigated the antioxidant activity of chokeberry juice phenolics, during in vitro simulated digestion, in the presence of food matrix. 

The antioxidant activity of carotenoids (lipophilic fractions) is based on the radical adducts of carotenoids with free radicals from linoleic acid. The linoleic acid free radical attacks the highly unsaturated β-carotene models. The presence of different antioxidants can hinder the extent of β-carotene bleaching by neutralizing the linoleate-free radical and other free radicals formed in the system [27]. Frankel and Mayer [28] reported that antioxidant activity may be explained in the basis of present antioxidant hydrophobicity and their solubility in linoleic acid emulsion. Hence, polar antioxidants are more active in bulk oil systems, whereas non-polar antioxidants are more active in lipids suspended in aqueous systems. This phenomenon was described by Frankel et al. [29] as a “polar paradox”, which proposes that non-polar antioxidants exhibit stronger antioxidant properties in oil water emulsions, because they become concentrated at the oil-water interface, thus protecting lipids from oxidation. The increase in antioxidant activity could be directly related to the released carotenoids during the digestive process, and therefore, FDE and SDE in SIF exhibited the highest values (50.51%, and 55.53%, respectively) followed by FDE and SDE in SGF (40.24%, and 31.56%, respectively). However, both tested encapsulates were showed lower activity than BHA at a level of 0.05 mg/mL (BCB% = 89.71 ± 0.11). 

**Figure 1:**
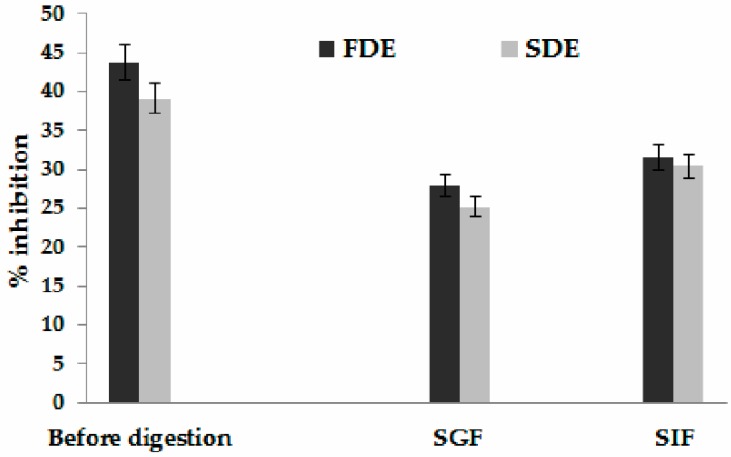
The potential of freeze-dried encapsulates (FDE) and spray-dried encapsulates (SDE)to inhibit α-glucosidase before and during simulated gastric (SGF) and intestinal fluids (SIF).

Type-II diabetes mellitus, generally characterized by insulin resistance, is a chronic metabolic disorder, and prevalence has been increasing steadily all over the world [30]. Of all available oral anti-diabetic drugs, α-glucosidase inhibitors regarded as the most effective therapeutic agent. However, the novel approaches to overcoming diabetes mellitus complications are aimed at avoiding synthetic drugs, due to its high price and considerable clinical side effects, so it is necessary to utilize naturally occurring α-glucosidase inhibitors. It is reported that plants have a great potential to retard the absorption of glucose by inhibiting the saccharides hydrolyzing enzymes, so in recent years their screening and isolation have escalated [1]. The potential of FDE and SDE to inhibit α-glucosidase is presented in Figure 1. The SIF exhibited the highest values with significant differences (*p* < 0.05) for FDE and SDE (31.6%, and 30.42%, respectively). Published research suggests that there is a direct relationship between phenolic compound, flavonoids, and condensed tannin in the plant extract and the ability to inhibit α-glucosidase activity [31,32]. Watcharachaisoponsiri et al. [33] investigated the α-glucosidase inhibitory activities of different commercially available chili peppers in Thailand. All investigated peppers inactivate α-glucosidase with different degrees of inhibition, within the range of 23–66% inhibition, where the red sweet pepper exhibited the highest inhibitory activity. Capsaicinoids are a group of bioactive compounds founded in chili peppers with pungent characteristic. However, peppers in *Capsicum annuum* species were reported to contain capsiate (non-pungent capsaicinoid) with a greater anti-diabetic action than capsaicin (pungent capsaicinoid) [34]. Ranila et al. [35] reported that quercetin and myricetin express α-glucosidase inhibitory effect as well.

**Figure 2:**
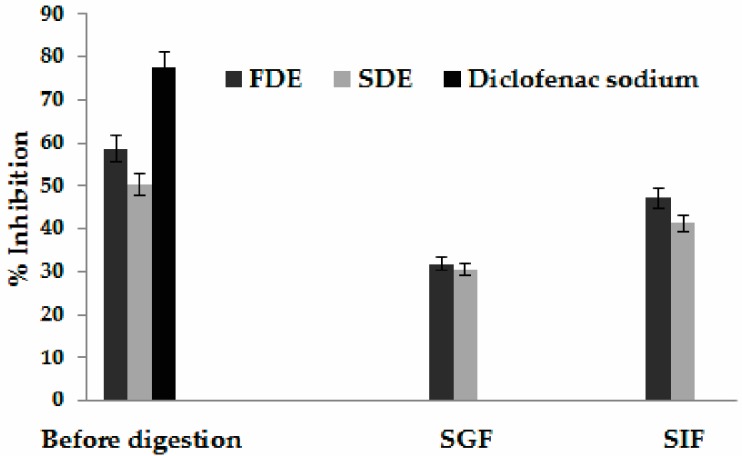
Anti-inflammatory activity of freeze-dried encapsulates (FDE) and spray-dried encapsulates (SDE) before and during simulated gastric (SGF) and intestinal fluids (SIF).

Inflammatory diseases include different types of rheumatic diseases, characterized by pain, swelling, and disturbed physiological functions. Protein denaturation is a well- documented cause of inflammation, followed by the loss of tertiary structure and secondary structure through the application of external stress or different compounds, as well as biological activities. The most commonly used drugs for the management of inflammatory conditions are non-steroidal anti-inflammatory drugs (NSAIDs). The greatest disadvantage in the presently available potent synthetic anti-inflammatory drugs lies in their toxicity, gastric irritation, thereby leading to the formation of gastric ulcers and the reappearance of symptoms after discontinuation [36]. Therefore, the search for natural bioactive compounds with anti-inflammatory activity has greatly increased in recent years. Hence, anti-denaturation of egg albumin method was chosen to evaluate anti-inflammatory activity FDE and SDE during simulated gastrointestinal digestion, where denaturation of egg albumin was induced by heat treatment (Figure 2). Diclofenac sodium was used as a positive control. Maximum inhibition of 47.13% was observed after SIF digestion of FDE, while minimum inhibition was observed after SGF of SDE (30.5%). However, both samples showed lower AIA than diclofenac sodium in concentration of 20 mg/mL (77.47%). There is significantly different values within FDE and SDE, before, and after, in vitro gastrointestinal digestion (*p* < 0.05). 

### 2.3. In Vitro Release Assay

**Figure 3:**
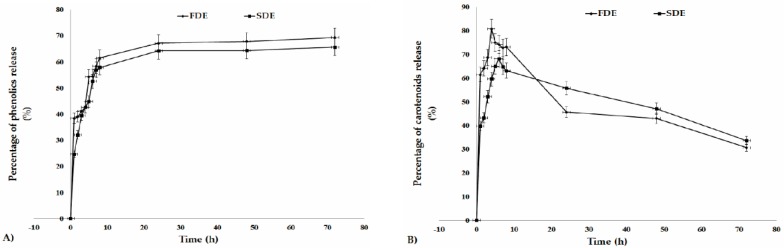
The release profile of phenolics (**A**) and β-carotene (**B**) from whey protein matrices under simulated conditions of the small intestine.

In order to determine whether the bioactive components are being released prematurely or gradually with time, the behavior of phenolics and carotenoids through the small intestine were simulated in vitro with a PBS at pH 7.4. The release profile of bioactive compounds from whey protein matrices, under simulated conditions of the small intestine, are presented in Figure 3. The gradual increase of phenolics release was observed, reaching around 50% for both samples during the first 6 h (Figure 3A). This observation can be associated with the solubility of the whey protein in the buffer. However, phenolics showed more stability than carotenoids toward the assay conditions. Over time, the percent of released β-carotene from FDE increased rapidly until 4 h, when the maximum of 80.79% was reached. Similar β-carotene release profile was observed for SDE, where 68.19% of β-carotene was released after 6 h (Figure 3B). This can be explained by the high dissolution rate of whey protein matrices in the reaction medium. However, it can be seen that there is a considerable reduction in β-carotene concentration for both samples. Incubation conditions (temperature, light exposure, and long duration of the assay) probably have a large impact on the oxidation and isomerization of carotenoids, due to their sensitivity toward exposed factors, and consequently on the degradation of these molecules. Similar carotenoid release behavior was reported by Sen Gupta and Ghosh [37] for fiber-encapsulated carotene nano-capsules. 

## 3. Material and Methods

### 3.1. Chemicals and Instruments

Folin-Ciocalteau reagent, 2,2-diphenyl-1-picrylhydrazyl radical (DPPH^●^), β-carotene, pancreatin, pepsin, Trolox and trichloroacetic acid were purchased from Sigma Chemical Co. (St Louis, MO, USA), ferric chloride was obtained from J.T. Baker (Deventer, Holland), and sodium nitrite from LACH-NER (Brno, Czech Republic). Other chemicals and solvents were of the highest analytical grade. Whey protein isolate (WPI) was purchased from Olimp Laboratories (Debica, Poland). Distilled water was produced using water purification system DESA 0081 Water Still destilator (POBEL, Madrid, Spain). Absorbance in spectrophotometrical assays was measured on a Multiskan GO microplate reader (Thermo Fisher Scientific Inc., Waltham, MA, USA). For HPLC analysis a Shimadzu Prominence chromatographic system was used, which consisted of LC-20AT binary pump, CTO-20A thermostat and SIL-20A autosampler connected to the SPD-20AV UV/Vis detector (Shimadzu, Kyoto, Japan). Freeze-dryer, model Christ Alpha 2-4 LSC, was from Martin Christ (Osterode am Harz, Germany), and spray-dryer (Buchi mini B-290) from BüchiLabortechnik AG (Flawil, Switzerland).

### 3.2. Plant Material

Fresh red pepper waste (RPW) material was obtained as by-product from the vegetable processing industry (“Zdravo Organic”, Selenča, Serbia). Waste material was freeze-dried, ground, packed in vacuumed plastic bags and stored at −20 °C until further analysis.

### 3.3. Extraction Procedure

Freeze-dried RPW (2.5 g) was extracted three times using acetone (50 mL) or acetone:ethanol mixture (50 mL; 36:64 *v*/*v*) in solid to solvent ratio 1:20 *w*/*v*, with the same volume of solvents. The extraction was performed using a laboratory shaker (Unimax 1010, Heidolph Instruments GmbH, Kelheim, Germany) at 300 rpm, under light protection, at room temperature, for 10 min. The obtained three extracts were filtered (Whatman paper No.1, Sigma-Aldrich, St. Louis, MO, USA), combined, and stored in dark bottles at −20 °C till further analysis. 

### 3.4. Encapsulation Process

Freeze-dried and spray-dried encapsulates were prepared following the method described by Šeregelj et al. [38] with some modifications. WPI (7 g) was dissolved in 10 mL of water at 60 °C and kept under stirring until the temperature reached 30 °C, while the mixture for spray-drying was dissolved in the same way in 40 mL of water. Separately, 40 mL of RPW extract was combined with sunflower oil (1.5 mL), concentrated under reduced pressure on a rotary evaporator set at 40 °C to remove the organic solvent, and immediately mixed with previously prepared carrier solution. The mixtures were homogenized at 11,000 rpm for 3 min at room temperature and subjected to freeze- and spray-drying.

#### 3.4.1. Freeze-Drying Conditions

The previously prepared mixture was iced overnight at −20 °C, then freeze-dried at −40 °C for 48 h to ensure complete drying. Collected FDE was stored at −20 °C until further use.

#### 3.4.2. Spray-Drying Conditions

The homogenized mixture was spray-dried using a laboratory scale spray-drier at an inlet temperature of 130 °C and an outlet temperature of 65 ± 2 °C. The spraying air flow rate and rate of liquid feed were 600 L/h and 8 mL/min, respectively. SDE was packed to zip-lock plastic bags and stored at −20 °C for later analyses.

### 3.5. In Vitro Simulated Gastrointestinal Digestion

In vitro digestion of encapsulates was determined by simulation of digestion in gastric fluid (SGF) and intestinal fluid (SIF), according to the method described by Vaštag et al. [39], with modifications. The first step of digestion was treating encapsulates (2.5% water suspension) with pepsin at pH 2.0 and 37 °C, during 1 h. After gastric digestion, the pH of the solution was adjusted to 7.5, pancreatin was added and the solution was stirred at same conditions during 2 h. At the end of each step of digestion, the sample aliquots were concentrated on a rotary evaporator and dissolved in methanol or hexane. In this way, lipophilic and hydrophilic fractions were obtained and analyzed for the bioavailability and bioactivity of encapsulated bioactive compounds isolated from RPW.

### 3.6. Bioavailability of Encapsulated Bioactive Compounds during In Vitro Simulated Gastrointestinal Digestion

The content of released phenolics and carotenoids in SGF and SIF were measured spectrophotometrically and by the HPLC method. The spectrophotometric analysis of total phenolics (TPh) in hydrophilic fractions was performed by Folin-Ciocalteau method, adapted to microscale. The results were expressed as gallic acid equivalents (GAE) per 100 g of encapsulates. Spectrophotometric analysis of total carotenoids (TCar) in lipophilic fractions was performed by the method of Nagata and Yamashita [40] and the results were expressed as mg of β-carotene equivalents per 100 g of encapsulates. For the HPLC analysis of phenolic compounds, two mobile phases, A (acetonitrile) and B (1% formic acid) were used at flow rates of 1 mL/min with the following gradient profile: 0–10 min from 10% to 25% A; 10–20 min linear rise up to 60% A, and from 20 min to 30 min linear rise up to 70% A, followed by 10 min reverse to initial 10% A with additional 5 min of equilibration time. Reference substances were dissolved in 50% methanol. Phenolic compounds were recorded using different wavelengths: 280 nm for hydroxybenzoic acids, 320 nm for hydroxycinnamic acids and 360 nm for flavonoids [41].

### 3.7. Bioactivity of Encapsulated Bioactive Compounds during In Vitro Simulated Gastrointestinal Digestion

The antioxidant activity, expressed as μmol Trolox equivalent (TE) per 100 g of encapsulates were performed by three methods: 2,2-diphenyl-1-picrylhydrazyl method (SA) described by Girones-Vilaplana et al. [42], reducing power (RP) by the Oyaizu [43], and β-carotene bleaching assay (BCB) by Al-Shaikan et al. [44]. The SA and RP were measured with hydrophilic fractions, while BCB was performed with hexane fractions. Antihyperglycemic activity (AHgA) was determined by measuring α-glucosidase inhibitory potential following the Tumbas Šaponjac et al. [41] method. In vitro assessment of anti-inflammatory activity (AIA) was determined by protein denaturation bioassay, according to method adopted by Ullah et al. [45]. Diclofenac sodium was used as a drug reference.

### 3.8. In Vitro Release Assay

In vitro carotenoids and phenolics release profiles from encapsulates were determined as reported by Kumar et al. [46], with some modifications. The encapsulates were re-dispersed in 0.01 mol/L phosphate-buffered saline solution (PBS; pH 7.4) at a final concentration 10 mg/mL. Eleven sets were incubated at 37 °C and at predetermined intervals of time aliquots were taken for measurement. The released carotenoids were redissolved in hexane, while phenolics were redissolved in methanol. Sample aliquots were analyzed for the amount of released carotenoids/phenolics as previously described and the released percentage was calculated according to the following equation:

Release (%) = ((Released carotenoids/phenolics)/(Total carotenoids/phenolics)) × 100(1)

### 3.9. Statistical Analysis

All experiments were run in triplicate. The results presented are means ± standard deviation (± SD, n = 3). Statistical analyses were carried out using Origin 8.0 SRO software package and Microsoft Office Excel 2010 software. Significant differences were calculated by ANOVA (*p* < 0.05).

## 4. Conclusions

Nowadays in society, there is a need for establish innovative technologies to reduce and reuse generated waste material, as a source of bioactive compounds with beneficial properties. Different encapsulation techniques may be used for preserving bioactive compounds obtained from food industry waste. However, encapsulation techniques may affect the physico-chemical characteristics and, thus, the quality of obtained encapsulates. In the present study, red pepper waste showed relatively high antioxidant capacity, reducing power, anti-hyperglycemic, and anti-inflammatory activity. Slightly better results were obtained for freeze drying than spray-drying technique. There was a rapid initial release of carotenoids from whey protein matrices, while a more gradual increase of phenolics was observed during simulated intestinal digestion than simulated gastric digestion. These results show that there is a big potential for pepper waste encapsulates as an antioxidant in food systems, due to its high content of phenolics, carotenoids, and antioxidant activity. Overall, the encapsulation of red pepper waste is efficient for functional food development, with improved nutrition, color, and bioactive properties.

## Figures and Tables

**Table 1:** Spectrophotometrical and HPLC analysis of bioactive compounds content before and after in vitro gastrointestinal digestion.

Compounds	Before Digestion		SGF		SIF	
	FDE	SDE	FDE	SDE	FDE	SDE
**Spectrophotometrical Analysis**						
**TPh ^a^**	1194.6 ± 0.9 ^b^	1183.1 ± 0.6 ^a^	642.4 ± 0.6 ^b^	433.6 ± 0.4 ^a^	742.1 ± 0.4 ^b^	673.1 ± 0.0 ^a^
**TCar ^b^**	4.5 ± 0.2 ^b^	3.2 ± 0.2 ^a^	0.6 ± 0.0 ^b^	0.3 ± 0.0 ^a^	0.9 ± 0.1 ^b^	0.5 ± 0.0 ^a^
**HPLC Analysis of Phenolics**						
**Gallic acid ^c^**	130.6 ± 1.2 ^a^	191.6 ± 1.2 ^b^	152.4 ± 1.4 ^b^	118.8 ± 1.1 ^a^	156.0 ± 2.8 ^b^	124.3 ± 1.7 ^a^
**Protocatechuic acid ^c^**	135.3 ± 0.7 ^b^	100.0 ± 0.1 ^a^	156.5 ± 1.9 ^b^	34.4 ± 0.0 ^a^	171.0 ± 0.9 ^b^	155.8 ± 1.3 ^a^
**Epicatechin ^c^**	63.6 ± 0.3 ^a^	68.3 ± 0.1 ^b^	34.4 ± 0.1 ^b^	14.2 ± 0.1 ^a^	25.0 ± 0.1 ^a^	33.7 ± 0.3 ^b^
**Chlorogenic acid ^c^**	10.4 ± 0.1 ^a^	-	-	-	-	-
**Vanillic acid ^c^**	43.7 ± 0.4 ^a^	-	23.6 ± 0.0 ^a^	--	17.1 ± 0.0 ^a^	-
**Caffeic acid ^c^**	4.9 ± 0.0 ^a^	15.0 ± 0.0 ^b^	0.4 ± 0.0 ^a^	1.0 ± 0.0 ^b^	0.4 ± 0.0 ^b^	0.1 ± 0.0 ^a^
**Myricetin ^c^**	2.7 ± 0.0 ^a^	6.7 ± 0.0 ^b^	0.3 ± 0.0 ^b^	0.2 ± 0.0 ^a^	0.3 ± 0.0 ^b^	0.1 ± 0.0 ^a^
**Quercetin ^c^**	-	-	0.3 ± 0.0 ^a^	-	0.3 ± 0.0 ^a^	-
**Rutin ^c^**	1.3 ± 0.0 ^a^	3.3 ± 0.0 ^b^	-	0.07 ± 0.00 ^a^	-	0.01 ± 0.0 ^a^
**Total phenolics ^c^**	392.5 ± 2.3 ^b^	384.9 ± 2.4 ^a^	367.9 ± 2.0 ^b^	168.7 ± 1.2 ^a^	370.1 ± 1.5 ^b^	314.0 ± 1.7 ^a^

SGF (simulated gastric fluid); SIF (simulated intestinal fluid); FDE (freeze-dried encapsulates); SDE (spray-dried encapsulates); data present mean value of three replicates ± SD; ^a^ Expressed as mg GAE/100 g; ^b^ Expressed as mg β-carotene/100 g; ^c^ Expressed as mg/100 g; Numbers sharing the same letter in row for each digestion step are not significantly different (*p* < 0.05).

**Table 2:** Antioxidant activity of FDE and SDE before and after in vitro gastrointestinal digestion.

Assay	Before Digestion		SGF		SIF	
	FDE	SDE	FDE	SDE	FDE	SDE
**SA ^a^**	1029.1 ± 44.6 ^b^	797.7 ± 28.6 ^a^	911.7 ± 60.8 ^b^	794.8 ± 8.8 ^a^	1304.4 ± 65.3 ^b^	1040.8 ± 20.5 ^a^
**RP ^a^**	1345.9 ± 6.2 ^b^	1055.1 ± 23.1 ^a^	796.4 ± 36.9 ^b^	750.5 ± 33.4 ^a^	1188.0 ± 29.8 ^b^	987.3 ± 16.0 ^a^
**BCB ^b^**	47.1 ± 1.8 ^b^	44.1 ± 2.2 ^a^	40.2 ± 1.9 ^b^	31.6 ± 1.4 ^a^	50.5 ± 2.8 ^a^	55.5 ± 2.3 ^b^

SA (free radical scavenging activity on DPPH radicals); RP (reducing power); BCB (β-carotene bleaching assay); Data present mean value of three replicates ± SD; ^a^ Expressed as µmol Trolox equivalents (TE)/100 g encapsulate; ^b^ Expressed as percent of inhibition relative to control; Numbers sharing the same letter in row for each digestion step are not significantly different (*p* < 0.05).
